# Disulfidptosis in heart failure: an emerging mechanism awaiting exploration

**DOI:** 10.3389/fcvm.2026.1842464

**Published:** 2026-09-09

**Authors:** Shuyi Ding, Yunke Zang, Yaqi Li, Wenjing Zhang, Zhong Liu, Chang Liu, Qiqi Wang, Liang Zhu, Kegang Ji

**Affiliations:** 1 Weifang Hospital of Traditional Chinese Medicine, Shandong Second Medical University, Weifang, China; 2 Department of Cardiovascular, Weifang Hospital of Traditional Chinese Medicine, Weifang, China; 3 School of Medical Laboratory, Shandong Second Medical University, Weifang, China; 4 College of First Clinical Medicine, Shandong University of Traditional Chinese Medicine, Jinan, China; 5 Department of Endocrinology and Metabolism, Affiliated Hospital of Shandong Second Medical University, Weifang, China; 6 Clinical Research Center, Affiliated Hospital of Shandong Second Medical University, Weifang, China

**Keywords:** disulfidptosis, F-actin, heart failure, NADPH, SLC7A11

## Abstract

Heart failure (HF) is a major cardiovascular syndrome with increasing global incidence and mortality rates. Cardiomyocyte injury and ventricular remodeling are central pathological processes driving HF progression. Although several forms of regulated cell death have been implicated in HF, the mechanisms underlying cardiomyocyte injury remain poorly understood. Disulfidptosis, proposed in 2023, is a metabolism-related form of regulated cell death triggered by the depletion of the cellular reducing capacity. Evidence from tumor cell models indicates that under glucose-restricted conditions, cells with high SLC7A11 expression may exhibit insufficient NADPH production due to impaired pentose phosphate pathway (PPP) activity, resulting in disulfide stress, aberrant disulfide cross-linking of filamentous actin (F-actin), cytoskeletal collapse, and cell death. During HF progression, cardiomyocytes commonly undergo glucose metabolic remodeling, redox imbalance, and cytoskeletal abnormalities, which may create a permissive context for disulfidptosis-like injuries. However, direct experimental evidence demonstrating disulfidptosis in cardiomyocytes or the failing myocardium remains limited. Rather than treating transcriptomic associations as evidence of a defined cell-death programme, this review integrates HF-associated metabolic remodelling, impaired redox buffering, and cytoskeletal vulnerability within a testable mechanistic framework. It further defines a staged validation strategy for determining whether disulfidptosis occurs in cardiomyocytes or other myocardial cell populations.

## 1 Introduction

Heart failure (HF) is a complex clinical syndrome that represents the final common pathway of many cardiovascular diseases. This has become a major global public health concern. In recent years, the prevalence of HF has continued to rise, with the global number of affected individuals estimated to exceed 56.2 million by 2021 (1). This growing burden is driven not only by population aging, but also by the increasing prevalence of metabolic risk factors, including diabetes, obesity, and metabolic syndrome (2, 3). Ventricular remodeling is a central pathological process in HF progression, characterized by myocardial hypertrophy, interstitial fibrosis, and persistent structural and functional alterations of cardiomyocytes (4, 5). Increasing evidence indicates that cardiomyocyte metabolic remodeling and the accompanying disruption of redox homeostasis play critical roles in the onset and progression of HF, serving as key molecular mechanisms underlying the progressive decline in cardiac function (6).

Disulfidptosis is a recently identified form of regulated cell death proposed in 2023. It is triggered by metabolic stress and is closely associated with the depletion of intracellular reducing capacity (7). Evidence from tumor cell models has shown that in cells with high SLC7A11 expression, glucose limitation or insufficient NADPH production impairs the reduction of imported cystine, leading to intracellular disulfide accumulation and disulfide stress (8). In these models, disulfide stress promotes aberrant disulfide cross-linking of cytoskeletal proteins, particularly F-actin, resulting in cytoskeletal collapse and subsequent cell death. Unlike apoptosis, ferroptosis, pyroptosis, or necroptosis, disulfidptosis is primarily characterized by the coupling of metabolic insufficiency, thiol-disulfide imbalance, and cytoskeletal disruption (9). However, whether this canonical mechanism operates in cardiomyocytes remains to be verified.

During HF progression, cardiomyocytes commonly exhibit impaired energy metabolism, altered glucose utilization, mitochondrial dysfunction, and disrupted redox homeostasis (10). These abnormalities partially overlap with the metabolic and redox conditions implicated in disulfidptosis, suggesting a possible biological link between disulfidptosis-related mechanisms and the failing myocardium.

Recent cardiovascular studies have applied predefined disulfidptosis-related gene sets to heart failure, hypertrophic cardiomyopathy, dilated cardiomyopathy, ischaemic cardiomyopathy, acute myocardial infarction, and atherosclerosis, primarily to identify differentially expressed genes, diagnostic markers, immune-associated patterns, or candidate therapeutic targets (11–17, 104, 105). These studies indicate that disulfidptosis-associated transcripts are altered across several cardiovascular conditions. However, transcriptomic association alone cannot determine whether the defining biochemical sequence of disulfidptosis occurs in diseased myocardium. In particular, it remains unclear whether SLC7A11-dependent cystine reduction becomes incompatible with NADPH availability and consequently leads to protein disulfide accumulation, actin disulfide cross-linking, and F-actin collapse. The unresolved question is therefore mechanistic rather than classificatory: whether HF creates the metabolic, redox, and structural conditions required for this pathway, and how such a process can be distinguished experimentally from ferroptosis and nonspecific oxidative injury.

Against this background, the present review extends the existing signature-oriented literature by organising available evidence within an HF-specific metabolism–redox–cytoskeleton framework. This framework links impaired substrate utilisation and NADPH generation to thiol–disulfide disequilibrium, actin-network vulnerability, and ventricular remodelling, while distinguishing established HF biology from mechanisms inferred mainly from tumour models. The exploratory transcriptomic analysis of GSE57338 is used to assess whether components of this framework are transcriptionally altered in failing myocardium (101, 102, 107); it is not intended to establish the occurrence of disulfidptosis or to derive another diagnostic gene signature. On this basis, we define the biochemical, structural, and rescue criteria required for causal validation and propose a staged experimental roadmap encompassing cell-type-resolved transcriptomics, metabolic and redox measurements, actin-focused structural analyses, and functional intervention studies in HF-relevant models.

## 2 Core mechanism of disulfidptosis: a brief overview

**Figure 1:**
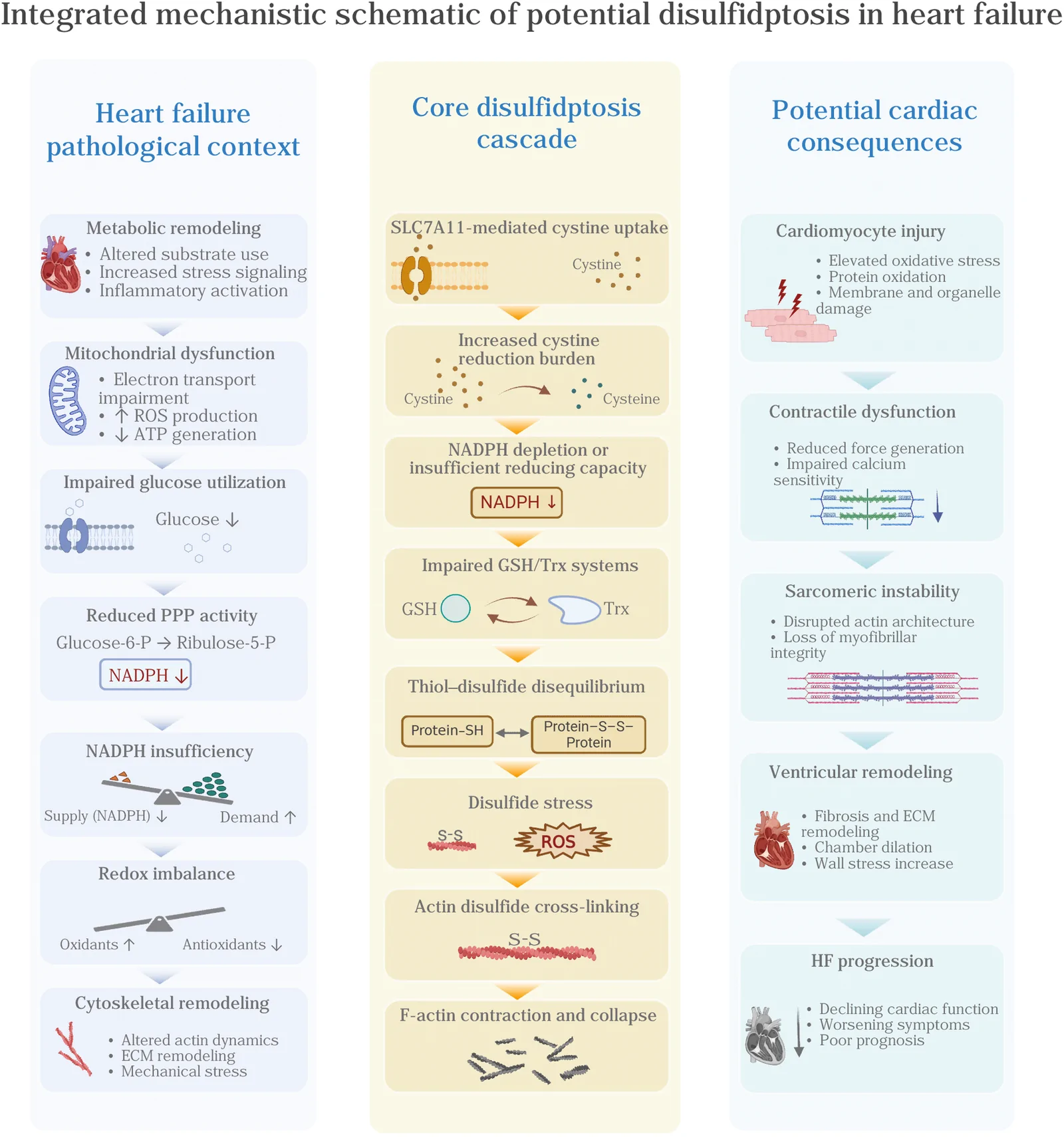
Integrated mechanistic schematic of potential disulfidptosis in heart failure. This schematic illustrates the proposed SLC7A11-NADPH-disulfide stress-F-actin collapse cascade in the pathological context of HF. HF-related metabolic remodeling, mitochondrial dysfunction, redox imbalance, and cytoskeletal instability may create a permissive environment for disulfidptosis-like injury.

To clarify this mechanistic link, we present an integrated schematic of the potential SLC7A11-NADPH-disulfide stress-F-actin collapse axis in HF (Figure 1). The figure outlines the core sequence of disulfidptosis and places it alongside key HF-associated pathological changes, including metabolic remodeling, mitochondrial dysfunction, impaired glucose utilization, reduced NADPH-generating capacity, redox imbalance, and cytoskeletal remodeling in HF.

### 2.1 Metabolic trigger: SLC7A11-mediated cystine uptake and NADPH depletion

Disulfidptosis is a recently proposed form of regulated cell death driven by a metabolic-redox imbalance. Its canonical mechanism has been defined mainly in tumor cells with high SLC7A11 expression under glucose-restricted conditions (8). As a cystine/glutamate antiporter, SLC7A11 provides cystine for intracellular cysteine production and glutathione synthesis, thereby supporting antioxidant defense (106) and protecting cells from oxidative injury and ferroptosis under sufficient metabolic conditions (18–20). However, sustained cystine uptake also increases the demand for NADPH-dependent reducing systems, particularly glutathione and thioredoxin(Trx) networks (21). When glucose availability is limited or pentose phosphate pathway activity is impaired, insufficient NADPH production may compromise thiol-buffering capacity and shift SLC7A11-mediated cystine uptake from an antioxidant-supporting process to a reduction burden that promotes the accumulation of disulfides (22, 23). This mechanism may be relevant to HF, in which cardiomyocytes commonly exhibit metabolic remodeling, mitochondrial dysfunction, impaired substrate utilization and redox imbalance (10). These changes can reduce the availability of NADPH and weaken thiol-disulfide homeostasis. Nevertheless, whether this SLC7A11-NADPH-dependent mechanism operates in cardiomyocytes under HF-related stress conditions remains to be clarified in future studies.

### 2.2 Biochemical basis: disulfide stress and thiol-disulfide imbalance

Disulfide stress occurs when disulfide bond formation exceeds the capacity of cellular reducing systems to reverse or repair these modifications (24). Under physiological conditions, protein cysteine thiols and disulfide bonds are maintained in a dynamic equilibrium by NADPH-dependent systems, particularly the glutathione and Trx networks (25–27). When NADPH supply becomes insufficient, these reducing systems may fail to preserve thiol-disulfide homeostasis, leading to the accumulation of aberrant disulfide bonds in proteins (28–31). In the context of disulfidptosis, this process should be distinguished from conventional oxidative stress. Whereas classical oxidative stress mainly refers to reactive oxygen species (ROS) mediated damage to lipids, proteins, and nucleic acids, disulfidptosis emphasizes impaired thiol-disulfide homeostasis and the structural consequences of abnormal protein disulfide bonding (32). This distinction is particularly relevant to HF research, as the failing myocardium is characterized not only by increased oxidative stress but also by impaired antioxidant capacity, altered reduced glutathione(GSH)/ oxidized glutathione(GSSG) balance, Trx dysfunction, and disturbed protein redox modifications (33–35). These abnormalities may create a biochemical environment compatible with disulfide stress, although whether they culminate in bona fide disulfidptosis in the failing myocardium remains to be clarified through further experimental validation.

### 2.3 Structural execution: F-actin collapse and potential relevance to HF

A defining structural event of disulfidptosis is the disruption of the actin cytoskeleton. In established non-cardiomyocyte models, particularly tumor cell models, insufficient reducing capacity promotes aberrant disulfide cross-linking of actin and actin-associated proteins, leading to contraction, disorganization, and collapse of the F-actin network (7, 36). This cytoskeleton-centered execution pattern distinguishes disulfidptosis from other forms of regulated cell death, such as apoptosis, ferroptosis, pyroptosis, and necroptosis, which are mainly driven by caspase activation, lipid peroxidation, inflammasome activation and membrane rupture (37, 38). This mechanism may be particularly relevant to cardiomyocytes because their highly organized actin-based sarcomeric structure is essential for contraction, force transmission, and maintaining mechanical stability (39–41). During HF, persistent mechanical overload, neurohormonal activation, mitochondrial dysfunction, and oxidative stress can disrupt cytoskeletal organization and modify the redox-sensitive cysteine residues in structural proteins (42). In this context, disulfide stress-induced cytoskeletal injury may provide a potential mechanistic link between metabolic-redox imbalance and contractile dysfunction in the failing myocardium (43, 44). However, current evidence for F-actin collapse during disulfidptosis is mainly derived from non-cardiomyocyte models. Whether cardiomyocytes develop SLC7A11-dependent disulfide stress, actin disulfide cross-linking, F-actin collapse, and disulfidptosis-like cell death under HF-related stress conditions remains to be determined in future studies.

## 3 Heart failure as a permissive pathological context for disulfidptosis

HF should not be interpreted as evidence of disulfidptosis in the myocardium. Instead, it represents a disease context in which the proposed SLC7A11-NADPH-disulfide stress-F-actin collapse cascade may be relevant. The convergence of metabolic remodeling, mitochondrial dysfunction, impaired reducing capacity, and cytoskeletal instability in the failing myocardium provides a rationale for exploring the disulfidptosis-like injury in HF (10).

### 3.1 Metabolic remodeling and NADPH insufficiency in HF

Metabolic remodeling is a hallmark of HF and may increase susceptibility to disulfidptosis-like injuries. Under physiological conditions, adult cardiomyocytes primarily rely on fatty acid oxidation to meet their high-energy demands. However, during HF progression, mitochondrial oxidative phosphorylation becomes impaired, substrate utilization becomes maladaptive, and glucose metabolism may no longer be sufficient to support both ATP production and redox homeostasis (45, 46). As NADPH is essential for glutathione- and Trx -dependent reducing systems, impaired NADPH-generating capacity may compromise the thiol-disulfide balance in cardiomyocytes (47, 100). In the canonical model of disulfidptosis, mainly established in tumor cell models, high SLC7A11-mediated cystine uptake increases the demand for NADPH-dependent reduction (8). When glucose metabolism or pentose phosphate pathway activity is impaired, this cystine reduction burden may exceed the cellular reducing capacity and promote disulfide stress. In failing myocardium, impaired glucose utilization and mitochondrial dysfunction can similarly reduce effective NADPH availability, thereby providing a metabolic rationale for exploring the disulfidptosis-like injury in HF (26, 45, 48). Whether NADPH depletion in cardiomyocytes reaches a sufficient level to engage this pathway remains an important question for future experimental studies.

### 3.2 Redox imbalance and thiol-disulfide disequilibrium in failing myocardium

Redox imbalance is a prominent pathological feature of HF. Mitochondrial dysfunction, neurohormonal activation, inflammation, and NADPH oxidase activation can increase ROS production in the failing myocardium (49, 50). Whereas antioxidant defenses may be weakened by impaired glutathione regeneration, Trx dysfunction, and insufficient NADPH supply (33). Together, these changes can promote cysteine oxidation and disturb the thiol-disulfide homeostasis (31). From the perspective of disulfidptosis, the key issue is not simply ROS accumulation but the inability of cellular reducing systems to reverse abnormal disulfide bond formation. Disulfide stress occurs when protein disulfide formation exceeds the repair capacity of glutathione, Trx, and related redox systems. In the failing myocardium, persistent oxidative stress and impaired reducing capacity may create a biochemical environment that favors disulfide stress (29, 33). However, whether these redox alterations progress to disulfidptosis in cardiomyocytes remains to be clarified by demonstrating protein disulfide accumulation, actin disulfide cross-linking, F-actin collapse, and SLC7A11-dependent cardiomyocyte death.

### 3.3 Cytoskeletal remodeling and structural vulnerability in HF

Cytoskeletal remodeling is a central feature of the structural progression of HF. During chronic pressure overload, ischemic injury, and neurohormonal stimulation, cardiomyocytes undergo sarcomeric reorganization, altered actin-myosin architecture, changes in titin stiffness, and remodeling of the cytoskeleton-associated signaling pathways (39, 51). Although these changes may serve as adaptive responses, they can become maladaptive as HF progresses.

This process is relevant to the discussion of disulfidptosis because cytoskeletal collapse is a major execution event in established experimental models, particularly tumor cell models. In failing myocardium, sustained mechanical stress, activation of mechanosensitive pathways such as RHOA/ROCK, PTK2/FAK, and YAP/TAZ, and redox-sensitive modifications of cytoskeletal proteins may increase the vulnerability of actin-based cardiomyocyte networks to oxidative stress (24, 52, 53). Unlike tumor cells, cardiomyocytes depend on highly ordered sarcomeres for contraction and mechanical coupling. Thus, disulfide stress-induced modifications of actin or actin-associated proteins could have functional consequences beyond cytoplasmic collapse, including impaired contractile performance, disrupted force transmission, and ventricular remodeling (40, 42). Whether this process contributes to F-actin disruption in failing cardiomyocytes remains an important question for future investigation.

### 3.4 SLC7A11 in HF: protective adaptation or metabolic vulnerability?

SLC7A11 may serve as a context-dependent regulatory node linking ferroptosis resistance to potential susceptibility to disulfidptosis. When the reducing capacity is sufficient, SLC7A11-mediated cystine uptake supports cysteine availability and glutathione synthesis, thereby strengthening antioxidant defense and suppressing ferroptosis (18, 54–56). However, under NADPH-limited conditions, sustained cystine uptake may impose an additional burden on cellular reducing systems and promote disulfide accumulation (57).

This dual role may be particularly relevant in patients with HF. During early adaptive stress responses, increased cystine uptake may help preserve glutathione-dependent antioxidant protection (55, 58, 59). In contrast, in advanced or decompensated HF, mitochondrial dysfunction, impaired glucose utilization, and reduced NADPH-generating capacity could shift SLC7A11 activity from a protective antioxidant mechanism toward a metabolic liability (48). This potential transition from redox protection to disulfide stress remains an important area for further investigation in cardiomyocytes and the failing myocardium.

This context dependence is also critical for distinguishing disulfidptosis from ferroptosis in HF. Ferroptosis has been implicated across chronic ischaemic, pressure-overload, diabetic, septic, obesity-related, and doxorubicin-induced cardiomyopathy models, in which disturbed iron handling, failure of the GSH–GPX4 antioxidant system, phospholipid peroxidation, and mitochondrial stress recur as major features (60). In these settings, insufficient system Xc^−^–GSH–GPX4 defence favours iron-dependent lipid peroxidation. Disulfidptosis represents a different metabolic outcome: when SLC7A11-mediated cystine uptake persists despite inadequate NADPH availability, cystine reduction may become a reductive burden that promotes protein disulfide accumulation and actin-network collapse. SLC7A11 expression alone therefore cannot determine which death programme, if either, is engaged.

Accordingly, future HF studies should assess SLC7A11 within its functional metabolic and redox context rather than as an isolated expression marker. Integrated measurements of the NADPH/NADP^+^ ratio, cystine/cysteine balance, GSH/GSSG ratio, GPX4 activity, labile iron, lipid peroxidation, protein disulfide accumulation, actin disulfide cross-linking, and F-actin integrity will be required to determine whether SLC7A11 supports antioxidant adaptation, promotes ferroptotic susceptibility, or contributes to disulfide stress under specific HF conditions. The core molecules involved in the proposed metabolic, redox, and cytoskeletal framework are summarized in Table 1.

**Table 1:** Core molecular components of the proposed disulfidptosis framework in heart failure and their evidence status.

Molecule/process	Proposed role in disulfidptosis	Evidence in HF	Evidence status	Ref.
SLC7A11/SLC3A2-system Xc^−^	Mediates cystine uptake and links cysteine availability, GSH synthesis, ferroptosis resistance, and the NADPH-dependent cystine-reduction burden.	Cardiac studies support a context-dependent role of SLC7A11 in antioxidant defence and ferroptosis. A consistent HF-wide direction for SLC7A11 or SLC3A2 has not been established. Direct involvement in myocardial disulfidptosis remains unproven.	Cardiac preclinical evidence for SLC7A11; exploratory transcriptomic evidence for HF-associated expression; no direct disulfidptosis evidence.	(12, 54, 55, 59)
NADPH-generating systems/PPP/G6PD	Supply reducing equivalents for cystine reduction and the GSH/Trx systems.	Impaired NADPH-dependent reducing capacity and altered PPP activity have been reported in HF-related settings, but direct NADPH abundance and G6PD activity vary with model, disease stage, and metabolic context.	Human and cardiac preclinical evidence for redox and metabolic remodelling; indirect evidence for disulfidptosis.	(22, 26, 47, 48)
Cystine/cysteine balance	Determines the cystine-reduction demand and provides substrate for GSH synthesis.	The myocardial cystine/cysteine balance has not been systematically defined across HF phenotypes. The direction of change cannot currently be assigned.	Mechanistic extrapolation; insufficient HF-specific evidence.	(7, 8, 18)
GSH/GSSG system	Maintains thiol reduction and supports GPX4-dependent lipid-peroxide detoxification.	Reduced GSH availability and impaired glutathione redox buffering have been reported in human and experimental HF, although the magnitude varies across models and disease stages.	Human and animal cardiac evidence; not specific to disulfidptosis.	(25, 26, 58)
Trx/TXNRD1 system	Uses NADPH to maintain protein thiols and repair abnormal disulfide bonds.	Alteration of the myocardial Trx system has been documented in human ischaemic cardiomyopathy. A universal direction or early-versus-late pattern has not been established.	Human cardiac evidence for redox dysregulation; indirect evidence for disulfidptosis.	(25, 33)
Protein disulfide accumulation	Represents the biochemical stress proposed to precede actin disulfide cross-linking.	Increased protein oxidation and intermolecular disulfide formation have been detected during cardiac oxidative stress and in failing human myocardium, but these changes are not specific to disulfidptosis.	Human cardiac and experimental evidence for disulfide stress; no pathway-specific evidence.	(28, 29, 35)
F-actin and the sarcomeric cytoskeleton	Actin disulfide cross-linking and F-actin collapse constitute the structural execution phase of canonical disulfidptosis.	Cytoskeletal and sarcomeric remodelling are established features of HF. However, disulfide-induced actin cross-linking and F-actin collapse have not been demonstrated as a death mechanism in failing myocardium.	Established HF cytoskeletal evidence; execution mechanism extrapolated mainly from non-cardiac systems.	(7, 36, 39–43)
Actin-remodelling genes: ACTB, CAPZB, MYH9, MYH10, MYL6, TLN1	Regulate actin organisation, myosin tension, adhesion, and force transmission.	Expression differences were identified in the exploratory myocardial transcriptomic analysis. These directions should not be interpreted as established molecular alterations across HF populations.	Exploratory bulk-transcriptomic evidence; external and cell-type-specific validation required.	(12)
Mitochondrial genes: NDUFS1, NDUFA11, NUBPL, LRPPRC	Influence respiratory-chain function, redox balance, and the availability of reducing equivalents.	Expression differences were identified in the exploratory analysis, but gene-specific directions have not been established consistently across HF cohorts.	Exploratory transcriptomic evidence only.	(12)
Rac1-WAVE/NCKAP1 signalling	Promotes actin polymerisation and may increase cytoskeletal susceptibility to disulfide stress.	Rac1-related signalling participates in cardiac oxidative and structural remodelling, whereas its role in myocardial disulfidptosis and the direction of NCKAP1 alteration remain unproven.	Cardiac pathway evidence plus non-cardiac mechanistic extrapolation.	(7, 42, 43)
Actin Cys374	Redox-sensitive cysteine residue involved in actin polymerisation and disulfide-mediated structural changes.	Redox regulation of Actin Cys374 is mechanistically established, but increased Cys374 disulfide cross-linking has not been directly demonstrated in human HF as evidence of disulfidptosis.	Non-HF mechanistic evidence; unvalidated in myocardial disulfidptosis.	(7, 36)

Human cardiac evidence indicates observations obtained from human failing myocardium or clinical cardiac samples. Cardiac preclinical evidence indicates findings from animal HF models or cultured cardiomyocytes. Exploratory transcriptomic evidence refers to associations identified in bulk-myocardial analysis and does not establish cell-type origin, protein-level alteration, or causal involvement. Extrapolated evidence refers to mechanisms derived predominantly from tumour or other non-cardiac systems. No molecular alteration listed here should be interpreted as proof that disulfidptosis occurs in HF.

G6PD, glucose-6-phosphate dehydrogenase; GPX4, glutathione peroxidase 4; GSH, reduced glutathione; GSSG, oxidised glutathione; HF, heart failure; NADPH, reduced nicotinamide adenine dinucleotide phosphate; PPP, pentose phosphate pathway; Trx, thioredoxin; TXNRD1, thioredoxin reductase 1.

## 4 Potential susceptibility of cardiomyocytes to disulfidptosis

Building on the HF-related pathological context discussed above, this section focuses on why cardiomyocytes may be particularly susceptible to disulfidptosis-like injuries. Unlike proliferating tumor cells, cardiomyocytes are terminally differentiated, highly energy-demanding, and structurally specialized cells that depend on mitochondrial metabolism, redox-buffering capacity, and sarcomeric integrity (10, 61). These features may influence the effects of metabolic–redox imbalance and disulfide stress on cardiomyocyte survival and function.

### 4.1 Metabolic dependence and NADPH vulnerability of cardiomyocytes

Cardiomyocytes are among the most energy-demanding cells in the body, requiring continuous mitochondrial ATP production and robust redox-buffering capacity to sustain contraction (61, 62). Under physiological conditions, adult cardiomyocytes predominantly rely on fatty acid oxidation and mitochondrial oxidative phosphorylation to meet their energy demands. During HF progression, however, mitochondrial dysfunction, chronic ischemia, altered substrate utilization, and impaired metabolic flexibility can disturb redox homeostasis (45, 63).

NADPH is essential for maintaining the glutathione- and Trx -dependent reducing systems. When NADPH availability declines, cardiomyocytes may become less capable of preserving protein thiols in a reduced state. In the canonical disulfidptosis model, primarily established in tumor cell systems, SLC7A11-mediated cystine uptake increases the demand for NADPH-dependent reduction (26). Thus, if cystine uptake remains active under conditions of impaired NADPH production, cardiomyocytes may experience excessive consumption of reducing equivalents and subsequent disulfide stress. This mechanism provides a metabolic rationale for considering disulfidptosis-like injury in failing cardiomyocytes, although its relevance in HF-specific models remains unclear.

### 4.2 Redox-sensitive protein networks in cardiomyocytes

Cardiomyocyte function relies on a wide range of redox-sensitive protein systems, including mitochondrial respiratory enzymes, calcium-handling proteins, sarcomeric proteins, and cytoskeletal regulators (29, 34, 64). Cysteine residues within these proteins can undergo reversible redox modifications, such as S-glutathionylation and disulfide bond formation, under physiological stress conditions (34, 65, 66). In HF, however, persistent oxidative stress and insufficient reducing capacity may shift these normally adaptive modifications toward maladaptive structural changes (33, 67).

From the perspective of disulfidptosis, this redox-sensitive protein network may be an important source of vulnerability. When glutathione, Trx, and protein disulfide repair systems are insufficient, abnormal disulfide bonds may accumulate and alter protein conformation, potentially affecting calcium handling, mitochondrial function, cytoskeletal stability, and contractile performance (68). Thus, the susceptibility of cardiomyocytes to disulfidptosis-like injury may depend not only on SLC7A11 expression, but also on the broader capacity of the cell to buffer thiol-disulfide stress. Whether these redox-sensitive protein alterations ultimately converge on disulfidptosis-specific cardiomyocyte death remains an important question for future investigations.

### 4.3 Sarcomeric and cytoskeletal specialization as a structural vulnerability

The structural specialization of cardiomyocytes distinguishes them from tumor cells and other cell types. Cardiomyocytes rely on highly organized sarcomeres and cytoskeletal networks to sustain contraction, mechanical coupling, and force transmission, with actin filaments, myosin, titin, α-actinin, desmin, and other structural proteins forming an integrated mechanical system essential for normal cardiac function (39, 69). This specialization may increase cardiomyocyte vulnerability to disulfide stress. In established non-cardiomyocyte models, aberrant disulfide cross-linking of actin cytoskeletal proteins during disulfidptosis leads to F-actin collapse (36). If a similar redox-dependent disruption of actin or actin-associated proteins occurs in cardiomyocytes, it could impair sarcomeric alignment, mechanical coupling, and contractile stability (68). Unlike tumor cells, in which F-actin collapse mainly causes cytoplasmic contraction and cell death, cardiomyocytes may experience broader functional consequences, including reduced force generation, impaired excitation-contraction coupling, and ventricular dysfunction (40). Whether this process occurs in the failing myocardium remains an important question for future investigations.

### 4.4 Stage-dependent role of SLC7A11 in cardiomyocytes

SLC7A11 may exert context-dependent effects on cardiomyocytes according to the underlying metabolic state. When the reducing capacity is preserved, SLC7A11-mediated cystine uptake supports cysteine availability and glutathione synthesis, thereby strengthening antioxidant defense and suppressing ferroptosis (18, 54, 68). This function may be protective during early adaptive responses to HF-related stress. In advanced HF, however, mitochondrial dysfunction, impaired glucose utilization, and reduced NADPH-generating capacity may limit the ability of cardiomyocytes to reduce imported cystine (48). Under these conditions, sustained SLC7A11 activity could increase NADPH consumption and promote disulfide accumulation, suggesting that SLC7A11 may act as a metabolic switch between redox protection and disulfide stress, depending on the disease stage and reducing capacity (57). This stage-dependent interpretation may help explain why SLC7A11-related pathways exert complex and even opposing effects across different HF contexts, although their precise roles in cardiomyocytes and failing myocardium remain an important question for future HF-relevant cellular and animal studies (59, 70).

## 5 Disulfidptosis in the network of cardiomyocyte death: crosstalk and distinct independence

**Figure 2:**
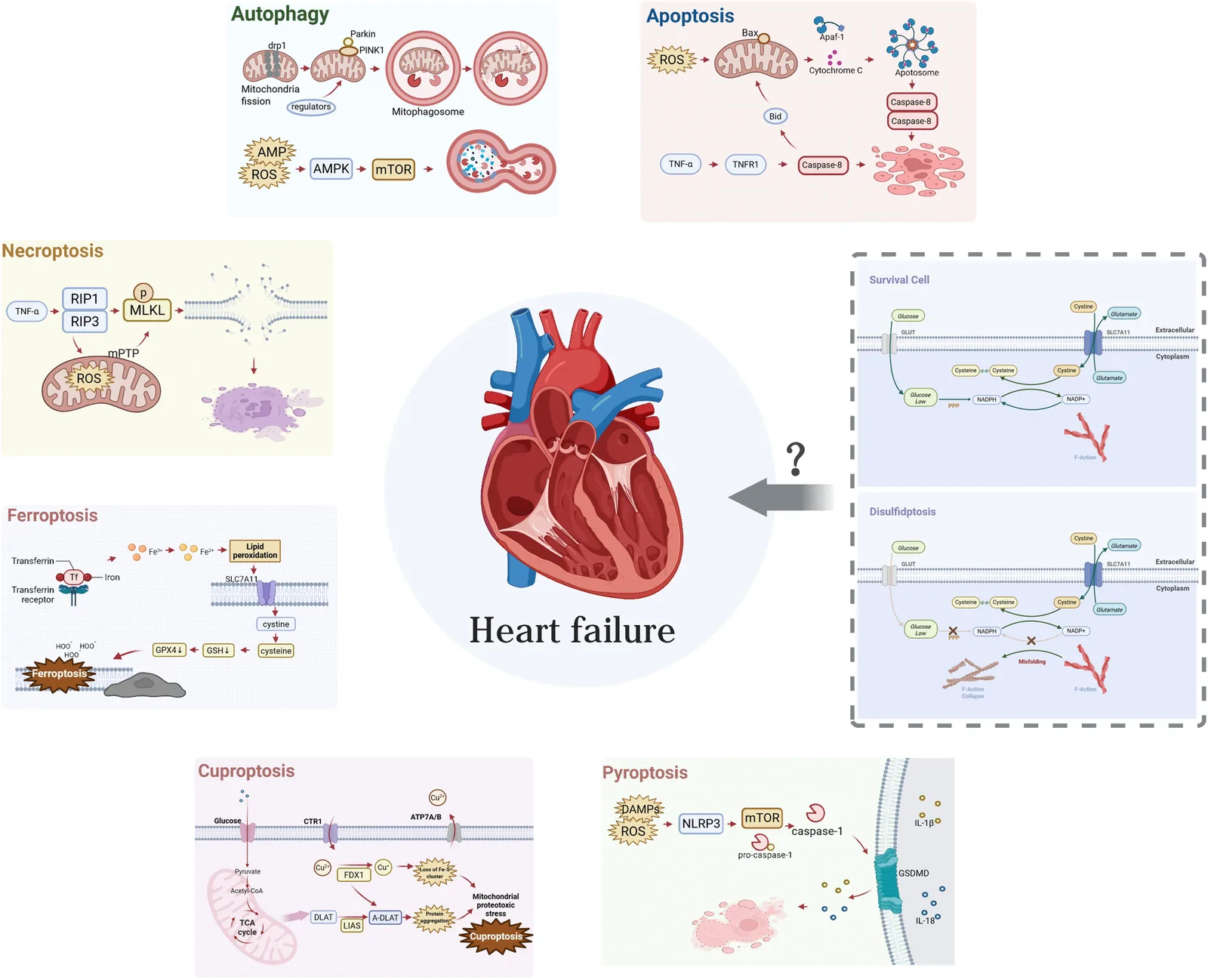
Different mechanisms of cardiomyocyte death. This figure summarizes major regulated cardiomyocyte death pathways involved in HF, including apoptosis, ferroptosis, pyroptosis, necroptosis, autophagy-dependent injury, cuproptosis and the predicted disulfidptosis pathway. Disulfidptosis may represent a cytoskeleton-centered injury mechanism linking SLC7A11-mediated cystine uptake, NADPH insufficiency, disulfide stress, and F-actin collapse. Its occurrence in cardiomyocytes and failing myocardium remains to be experimentally validated.

Cardiomyocyte death in HF arises from an interconnected network of regulated cell death pathways rather than from a single isolated mechanism. Apoptosis, ferroptosis, pyroptosis, necroptosis, autophagy-dependent injury, cuproptosis, and other emerging forms of cell death may coexist or interact under ischemic stress, pressure overload, neurohormonal activation, mitochondrial dysfunction, inflammation, and metabolic stress (37, 71, 72). Within this complex landscape, disulfidptosis may represent a distinct but related mode of injury that links metabolic insufficiency, redox failure, and cytoskeletal collapse. Its proposed position within the broader framework of regulated cardiomyocyte death in HF is illustrated in Figure 2.

Rather than reviewing each regulated cell death pathway in detail, this section focuses on the crosstalk and mechanistic distinctions between disulfidptosis and other death programs in HF. Although these pathways may share upstream stressors, such as metabolic dysfunction, oxidative stress, inflammation, and impaired reduction capacity, disulfidptosis is distinguished by abnormal disulfide bond accumulation, actin disulfide cross-linking, and F-actin collapse. This cytoskeleton-centered execution pattern may be particularly relevant to cardiomyocytes, where sarcomeric integrity is essential for contraction and ventricular function.

### 5.1 Crosstalk with apoptosis

Apoptosis is a classical form of regulated cardiomyocyte death in HF, characterized by mitochondrial outer membrane permeabilization, cytochrome c release, caspase activation, and DNA fragmentation (73, 74). Its potential crosstalk with disulfidptosis may occur mainly at the level of shared upstream stressors, such as mitochondrial dysfunction, ATP deficiency, oxidative stress, and impaired reducing capacity. However, the two pathways diverge markedly in their mechanisms of action. Apoptosis proceeds through caspase-mediated proteolysis and nuclear fragmentation, whereas disulfidptosis centers on disulfide stress-induced collapse of the actin cytoskeleton. In this sense, disulfidptosis may represent a cytoskeleton-centered injury process that is not fully captured by classical apoptotic pathways.

### 5.2 Crosstalk with ferroptosis

Ferroptosis is mechanistically close to disulfidptosis because both are influenced by the SLC7A11–GSH–NADPH axis. In ferroptosis, SLC7A11-mediated cystine uptake supports cysteine availability, GSH synthesis, and GPX4-dependent detoxification of phospholipid hydroperoxides. Impairment of this defence permits iron-dependent peroxidation of polyunsaturated phospholipids and subsequent membrane injury. Ferroptosis-related changes have been reported across several HF-relevant models, including chronic ischaemic injury, pressure overload, diabetic cardiomyopathy, septic and obesity-related cardiac dysfunction, and doxorubicin-induced cardiomyopathy (60, 103). These studies provide a substantially broader HF-specific experimental basis for ferroptosis than is currently available for disulfidptosis.

The overlap at SLC7A11 does not imply a shared execution mechanism. In the canonical disulfidptosis model, persistent cystine uptake under NADPH-limited conditions increases the demand for cystine reduction and promotes protein disulfide stress. The principal downstream event is abnormal disulfide cross-linking of actin and actin-associated proteins, followed by contraction and collapse of the F-actin network. Ferroptosis, by contrast, is centred on iron-dependent phospholipid peroxidation and membrane damage. Pathway assignment in HF should therefore not rely on SLC7A11 expression, GSH depletion, or oxidative stress alone. Evidence for ferroptosis should include iron and lipid-peroxidation measurements, disruption of the GPX4-centred defence system, and rescue by ferroptosis inhibitors or iron chelation. Evidence for disulfidptosis should include NADPH insufficiency, protein and actin disulfide accumulation, F-actin collapse, and rescue through restoration of reducing capacity or reduction of the cystine burden.

### 5.3 Crosstalk with pyroptosis and necroptosis

Pyroptosis and necroptosis are inflammatory and necrotic forms of regulated cell death that contribute to inflammatory amplification and adverse remodeling in HF (71, 75). Pyroptosis is characterized by inflammasome activation, gasdermin-mediated pore formation, and the release of IL-1β and IL-18, whereas necroptosis is primarily mediated by the RIPK1-RIPK3-MLKL axis and culminates in membrane rupture (76). Their potential crosstalk with disulfidptosis may occur through shared upstream processes, including mitochondrial ROS generation, danger-associated molecular pattern release, inflammatory signaling, and impaired antioxidant capacity (77). However, their execution mechanisms are different. Pyroptosis and necroptosis ultimately lead to pore formation or membrane rupture, whereas disulfidptosis centers on thiol-disulfide imbalance and actin cytoskeletal collapse.

### 5.4 Crosstalk with autophagy-dependent injury

Autophagy is a homeostatic process responsible for removing damaged organelles and misfolded proteins. In HF, adaptive autophagy and mitophagy reduce mitochondrial oxidative stress and support cardiomyocyte survival, whereas excessive autophagy or impaired autophagic flux may aggravate cellular injury (78–80). Therefore, its relationship with disulfidptosis may be bidirectional. Efficient autophagic clearance can limit disulfide stress by removing damaged mitochondria and oxidized proteins, while defective autophagic flux may intensify redox imbalance and cytoskeletal instability (43). Nevertheless, autophagy-dependent injury is mainly associated with lysosomal degradation and impaired cellular clearance, whereas disulfidptosis is characterized by disulfide stress-induced collapse of the actin cytoskeleton.

### 5.5 Crosstalk with cuproptosis

Cuproptosis is an emerging form of regulated cell death associated with copper-dependent mitochondrial proteotoxic stress and the aggregation of lipoylated tricarboxylic acid cycle proteins (72). Its relationship with disulfidptosis mainly reflects a shared metabolic vulnerability rather than a direct mechanistic overlap. Both pathways illustrate how metabolic disturbances can trigger non-classical cell death programs. However, their execution mechanisms differ. Cuproptosis is driven by copper-induced mitochondrial protein aggregation, whereas disulfidptosis is characterized by NADPH insufficiency, thiol-disulfide disequilibrium, and actin cytoskeletal collapse. Therefore, cuproptosis is mentioned here only as a metabolism-related comparator, rather than being discussed as a major focus of this review.

### 5.6 Integrated perspective

Taken together, disulfidptosis should be placed within the broader network of cardiomyocyte death while retaining its distinct mechanistic identity. Its crosstalk with other death pathways mainly arises from shared upstream stressors, including mitochondrial dysfunction, oxidative stress, metabolic insufficiency, ATP depletion, inflammation, calcium dysregulation, and impaired antioxidant capacities (37). In contrast, its mechanistic specificity lies in its downstream processes: protein disulfide accumulation, actin disulfide cross-linking, and F-actin collapse (7). This feature distinguishes disulfidptosis from apoptosis, ferroptosis, pyroptosis, necroptosis, autophagy-dependent injury, and cuproptosis. Future studies should determine whether disulfidptosis occurs as an independent form of cardiomyocyte death or functions as part of a broader metabolic-redox injury network in HF. The major crosstalk and mechanistic distinctions between disulfidptosis and other cardiomyocyte death pathways in HF are summarized in Table 2, with a more detailed comparison provided in Supplementary Table S1.

**Table 2:** Comparison of disulfidptosis with major cardiomyocyte death pathways in HF.

Cell death mode	Main upstream triggers in HF	Core execution mechanism	Relationship with disulfidptosis	Key distinction
Apoptosis	Mitochondrial dysfunction, ischemia, oxidative stress, ER stress	Cytochrome c release, caspase activation, DNA fragmentation	Shares mitochondrial stress and ATP deficiency	Caspase-dependent proteolysis rather than disulfide stress-induced F-actin collapse
Ferroptosis	Iron overload, GSH depletion, GPX4 dysfunction, lipid peroxidation	Iron-dependent lipid peroxide accumulation	Shares SLC7A11-GSH-NADPH axis	Lipid peroxidation is central, whereas disulfidptosis centers on protein disulfide accumulation and cytoskeletal collapse
Pyroptosis	Inflammation, NLRP3 activation, mitochondrial ROS	Caspase-1/GSDMD pore formation and IL-1β/IL-18 release	Inflammatory ROS may weaken redox buffering	Inflammatory pore-forming death rather than actin disulfide cross-linking
Necroptosis	TNF signaling, oxidative stress, calcium overload	RIPK1/RIPK3/MLKL-mediated membrane rupture	Shares upstream oxidative and mitochondrial stress	MLKL-mediated membrane disruption rather than F-actin collapse
Autophagy-dependent injury	Energy stress, impaired mitophagy, AMPK-mTOR dysregulation	Excessive or defective lysosomal degradation	Autophagy may buffer or amplify disulfide stress	Lysosomal degradation rather than disulfide-driven cytoskeletal collapse
Disulfidptosis	NADPH insufficiency, SLC7A11-mediated cystine burden, thiol–disulfide imbalance	Protein disulfide accumulation, actin disulfide cross-linking, F-actin collapse	Intersects with metabolic-redox stress networks	Unique dependence on disulfide stress-induced cytoskeletal collapse

## 6 Exploratory transcriptomic analysis

To examine whether the proposed metabolism–redox–cytoskeleton framework is transcriptionally altered in human HF, we analysed three primary myocardial RNA-sequencing cohorts (GSE116250, GSE55296, and GSE135055) and used GSE57338 as an independent cross-platform validation cohort. Each dataset was processed and analysed separately. The analysis was designed to assess reproducible gene-level and module-level transcriptional patterns, rather than to derive a diagnostic classifier or infer the occurrence of disulfidptosis from transcript abundance.

### 6.1 Materials and methods

#### 6.1.1 Dataset source and cohort composition

Three human myocardial RNA-sequencing cohorts were used for the primary analysis: GSE116250 (14 normal and 37 HF samples), GSE55296 (10 normal and 13 HF samples), and GSE135055 (9 normal and 18 HF samples). GSE57338 served as the independent cross-platform validation cohort and comprised 313 left-ventricular myocardial samples generated on the GPL11532 Affymetrix Human Gene 1.1 ST Array, including 136 non-failing donor hearts and 177 HF samples (95 ischaemic cardiomyopathy and 82 idiopathic dilated cardiomyopathy) (80). HF and normal samples were compared within each cohort; expression matrices and individual-level data were not pooled across platforms.

#### 6.1.2 Expression-data preprocessing and gene annotation

Processed log_2_-scale expression matrices were used for all four cohorts, and no additional logarithmic transformation was applied. Gene identifiers were standardised to uppercase official gene symbols, entries without valid symbols were removed, and duplicate symbols were collapsed by mean expression using limma::avereps. Sample names were matched to the corresponding grouping files before analysis. Each cohort was normalised, scored, and tested independently; no direct matrix merging, cross-platform batch correction, or ComBat adjustment was performed.

#### 6.1.3 Candidate-gene panel and evidence classification

**Figure 3:**
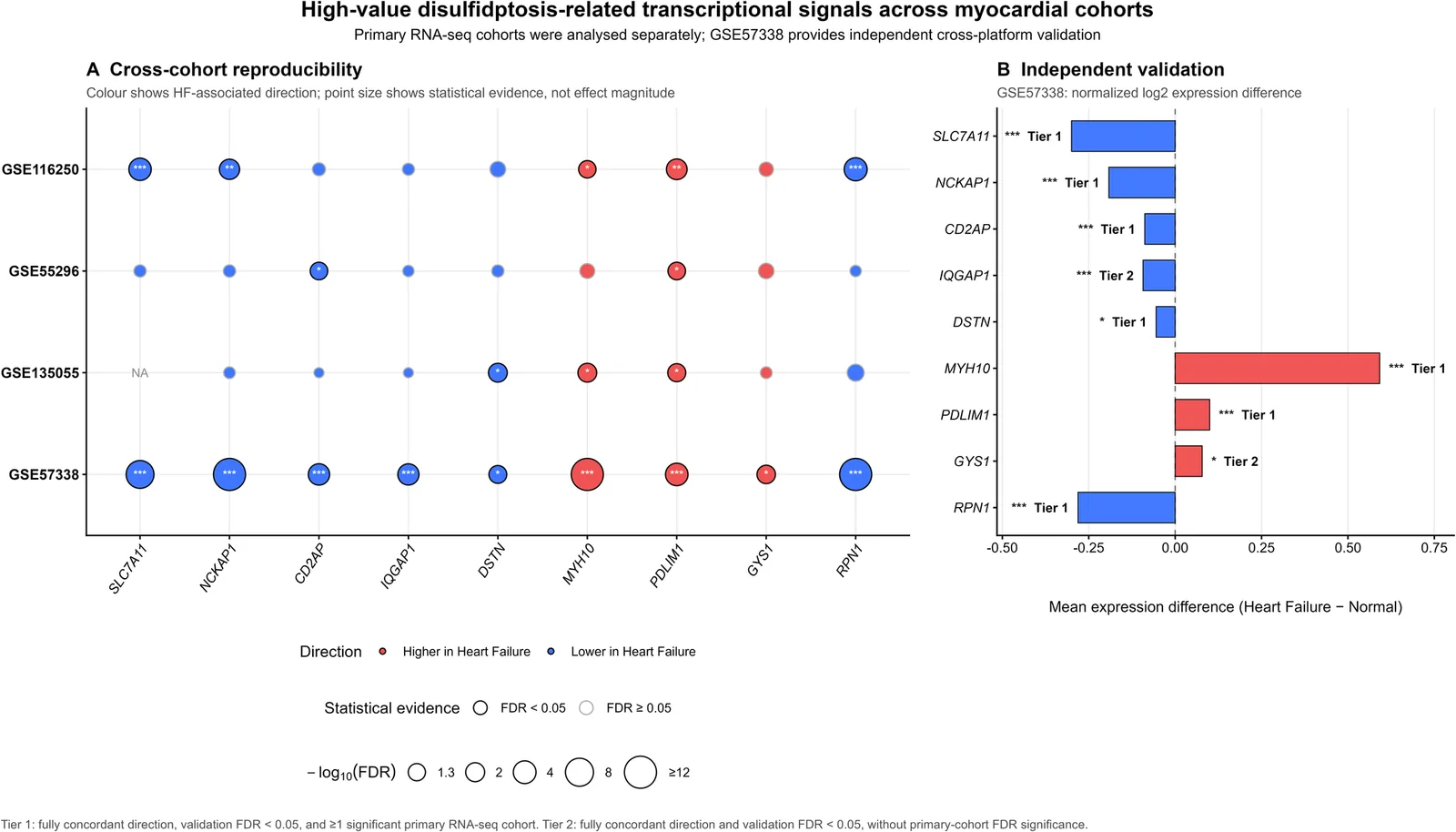
Cross-cohort reproducibility and independent validation of high-value disulfidptosis-related transcriptional signals in failing myocardium. **(A)** Cross-cohort expression patterns of nine candidate genes across three primary RNA-sequencing cohorts (GSE116250, GSE55296, and GSE135055) and the independent validation cohort GSE57338. Each cohort was analysed separately. Red and blue circles indicate higher and lower expression, respectively, in heart failure (HF) relative to normal myocardium. Circle size is proportional to −log_10_(FDR) and therefore represents the strength of statistical evidence rather than the magnitude of the expression difference. Black circle borders indicate FDR < 0.05, whereas grey borders indicate FDR ≥ 0.05. “NA” indicates that the corresponding gene was unavailable or could not be evaluated in that cohort. **(B)** Independent cross-platform validation in GSE57338. Horizontal bars represent the mean normalized log_2_ expression difference between HF and normal myocardium (HF−Normal). Positive red bars indicate higher expression in HF, whereas negative blue bars indicate lower expression in HF. Tier 1 genes were defined as genes showing concordant expression direction across all evaluable cohorts, an FDR < 0.05 in GSE57338, and an FDR < 0.05 in at least one primary RNA-sequencing cohort. Tier 2 genes showed concordant direction across all evaluable cohorts and an FDR < 0.05 in GSE57338 but did not reach FDR significance in any primary RNA-sequencing cohort. Asterisks denote statistical significance: *FDR < 0.05, **FDR < 0.01, and ***FDR < 0.001. FDR, false discovery rate; HF, heart failure.

The cross-cohort candidate panel shown in Figure 3 comprised nine genes: SLC7A11, NCKAP1, CD2AP, IQGAP1, DSTN, MYH10, PDLIM1, GYS1, and RPN1. Evidence classification was based on the concordance of HF-associated expression direction across all evaluable cohorts and on independent validation in GSE57338. Tier 1 genes showed fully concordant direction, an FDR below 0.05 in GSE57338, and an FDR below 0.05 in at least one primary RNA-sequencing cohort. Tier 2 genes showed fully concordant direction and an FDR below 0.05 in GSE57338, but did not reach FDR significance in any primary cohort.

#### 6.1.4 Cohort-specific gene-level analysis

For each cohort and candidate gene, expression distributions in HF and normal myocardium were compared using two-sided Wilcoxon rank-sum tests. *P* values were adjusted within each cohort across the evaluated candidate panel using the Benjamini–Hochberg method. The expression direction was defined from the difference in mean normalised log_2_ expression (HF minus normal), whereas statistical evidence was represented by −log_10_(FDR). An FDR below 0.05 was considered significant.

#### 6.1.5 Definition and scoring of the molecular modules

Three study-specific gene sets were defined to represent the principal components of the proposed framework: an NADPH-generating metabolic programme (10 genes), a redox-buffering programme (15 genes), and an actin-cytoskeletal-remodelling programme (23 genes). Their complete membership and source rationale are provided in Supplementary Table S2. Within each cohort, expression of every available, non-zero-variance gene was *Z*-standardised across samples, and the sample-level module score was calculated as the mean *Z* score of the genes in the corresponding set. The final module score was standardised within each cohort and module to provide a common visual scale. This procedure was implemented with custom R code and was not an ssGSEA or GSVA analysis.

HF and normal module scores were compared separately in each cohort using two-sided Wilcoxon rank-sum tests. Benjamini–Hochberg correction was applied globally across the 12 displayed comparisons (four cohorts by three modules). These expression-derived scores were interpreted as relative transcriptional programmes rather than direct measurements of NADPH concentration, antioxidant capacity, protein-disulfide accumulation, or cytoskeletal integrity.

#### 6.1.6 HF-related comparator pathways and cross-cohort correlation meta-analysis

Ten study-specific HF comparator pathways were evaluated: oxidative stress, mitochondrial dysfunction, oxidative phosphorylation, cardiac muscle contraction, actin-filament organisation, extracellular-matrix organisation, TGF-β signalling, inflammatory response, ferroptosis, and apoptosis. Exact gene-set membership and source information are listed in Supplementary Table S3. Correlations were calculated within HF samples only. For each signature–pathway pair, genes shared by the disulfidptosis-related module and comparator pathway were removed from the pathway score before analysis to reduce circular correlation caused by overlapping membership. Two-sided Spearman correlations were then estimated separately in each cohort. Cohort-specific correlation coefficients were transformed using Fisher *z* values and combined with random-effects models estimated by restricted maximum likelihood.

#### 6.1.7 Statistical software and reproducibility

All analyses were performed in R version [4.4.3]. GEO data processing and differential-expression analyses were conducted using GEOquery version [2.74.0] and limma version [3.62.2], respectively. Transcriptional programme scores were calculated using custom R scripts based on the mean *Z*-standardized expression of the available genes within each predefined gene set. Correlation analyses and random-effects meta-analyses were performed using the stats and metafor packages, respectively. Figures were generated using ggplot2, ggpubr, and pheatmap. The analysis code, sample-grouping information, gene-set definitions, package-session information, and complete statistical results are provided in the Supplementary Materials.

### 6.2 Results

#### 6.2.1 Cross-cohort reproducibility of candidate genes

The nine candidate genes showed concordant HF-associated directions across all evaluable cohorts and were independently significant in GSE57338 (Figure 3). SLC7A11, NCKAP1, CD2AP, IQGAP1, DSTN, and RPN1 were lower in HF, whereas MYH10, PDLIM1, and GYS1 were higher. SLC7A11 was not evaluable in GSE135055 because its expression was invariant in that matrix. In the primary cohorts, SLC7A11, NCKAP1, MYH10, PDLIM1, and RPN1 reached FDR significance in GSE116250; CD2AP and PDLIM1 in GSE55296; and DSTN, MYH10, and PDLIM1 in GSE135055.

Seven genes—SLC7A11, NCKAP1, CD2AP, DSTN, MYH10, PDLIM1, and RPN1—met the Tier 1 criteria, whereas IQGAP1 and GYS1 were classified as Tier 2. These results identify reproducible bulk-myocardial transcriptional signals, but they do not establish their cellular origin or show that the corresponding proteins participate in a disulfidptosis cascade in cardiomyocytes (Supplementary Table S4).

#### 6.2.2 Alterations in metabolic, redox, and cytoskeletal module scores

**Figure 4:**
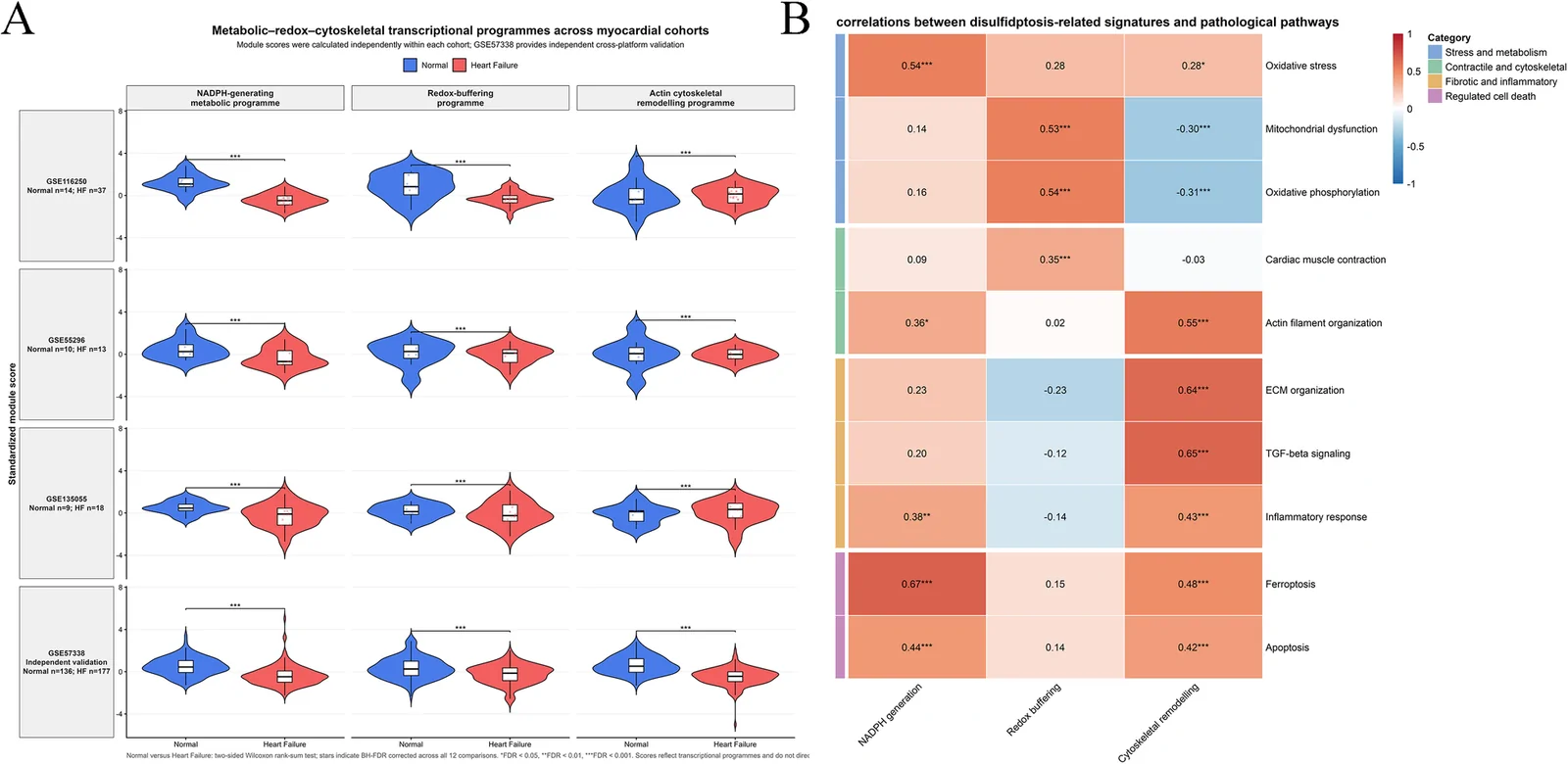
Metabolic–redox–cytoskeletal transcriptional programmes in heart failure. **(A)** Scores of the NADPH-generating, redox-buffering, and actin-cytoskeletal programmes in normal and heart-failure myocardium across four cohorts. Scores were calculated independently within each cohort using mean gene *Z* scores. Groups were compared by two-sided Wilcoxon rank-sum tests with Benjamini–Hochberg correction across 12 comparisons. **(B)** Meta-analysed Spearman correlations between the three programmes and heart-failure pathological pathways. Correlations were calculated within HF samples after removing overlapping genes and combined using random-effects models after Fisher *z* transformation. Red and blue indicate positive and negative correlations, respectively. FDR < 0.05, FDR < 0.01, FDR < 0.001.

All 12 HF-versus-normal module comparisons remained significant after global Benjamini–Hochberg correction (all FDR < 0.001; Figure 4A). The NADPH-generating and redox-buffering programme scores were lower in HF in all four cohorts. By contrast, the actin-cytoskeletal-remodelling score was higher in HF in GSE116250 and GSE135055 but lower in GSE55296 and GSE57338, indicating directionally heterogeneous cytoskeletal remodelling across cohorts. Thus, the metabolic and redox programmes showed consistent attenuation, whereas the cytoskeletal programme reflected context-dependent transcriptional reorganisation rather than uniform suppression or activation.

#### 6.2.3 Cross-cohort associations with HF pathological pathways

Random-effects meta-analysis of within-HF correlations identified distinct association patterns (Figure 4B). The NADPH-generating programme was positively associated with oxidative stress (pooled *ρ* = 0.54), actin-filament organisation (*ρ* = 0.36), inflammatory response (*ρ* = 0.38), ferroptosis (*ρ* = 0.67), and apoptosis (*ρ* = 0.44). The redox-buffering programme was positively associated with mitochondrial dysfunction (*ρ* = 0.53), oxidative phosphorylation (*ρ* = 0.54), and cardiac muscle contraction (*ρ* = 0.35).

The cytoskeletal-remodelling programme was positively associated with oxidative stress (*ρ* = 0.28), actin-filament organisation (*ρ* = 0.55), extracellular-matrix organisation (*ρ* = 0.64), TGF-β signalling (*ρ* = 0.65), inflammatory response (*ρ* = 0.43), ferroptosis (*ρ* = 0.48), and apoptosis (*ρ* = 0.42), but was negatively associated with mitochondrial dysfunction (*ρ* = −0.30) and oxidative phosphorylation (*ρ* = −0.31). All cited associations remained significant after FDR correction. Because these values represent pooled correlations between expression-derived scores, even after pairwise removal of overlapping genes, they indicate cross-cohort transcriptomic co-variation rather than direct pathway interaction or causal coupling. Taken together, the findings support coordinated but non-sequential alterations across the metabolic, redox, and cytoskeletal domains; they do not demonstrate activation of an SLC7A11–NADPH–disulfide stress–F-actin collapse cascade in failing myocardium (Supplementary Table S5).

### 6.3 Limitations of the transcriptomic analysis

Several limitations should be considered when interpreting these findings.

#### 6.3.1 Bulk-tissue composition and cell-type origin

The four datasets were generated from bulk left-ventricular tissue. The observed expression patterns therefore represent composite signals from cardiomyocytes, fibroblasts, endothelial and vascular cells, and resident or infiltrating immune populations, and may also reflect disease-associated shifts in cellular composition. Accordingly, the candidate-gene, redox, and cytoskeletal signals cannot be assigned specifically to cardiomyocytes.

This caveat is supported by recent single-cell evidence from human dilated cardiomyopathy. Karampinos et al. identified a ferroptosis-associated transcriptional programme that was enriched predominantly in fibroblasts and associated with progressive fibroblast activation and profibrotic remodelling. Given that ferroptosis and disulfidptosis intersect at the SLC7A11–GSH–NADPH regulatory axis, it is plausible that part of the redox-related bulk signal detected here arises from stromal compartments. This interpretation remains inferential: a fibroblast-enriched ferroptosis-associated signature does not demonstrate fibroblast disulfidptosis, nor does it establish that the same cellular distribution applies to the present gene modules.

#### 6.3.2 Aetiological heterogeneity and external validation

Although the use of three primary cohorts and an independent cross-platform validation cohort improves reproducibility, the datasets differ in sample size, platform, HF aetiology, and clinical composition. In particular, GSE57338 combines ischaemic and idiopathic dilated cardiomyopathy. The available metadata did not support uniform stratification by aetiology, disease stage, sex, age, or treatment across all cohorts. These differences may contribute to the heterogeneous cytoskeletal results and limit direct biological comparison of effect magnitude between datasets.

#### 6.3.3 Co-derivation of expression scores and gene-set overlap

Within each cohort, the molecular-module and comparator-pathway scores were calculated from the same expression matrix. Pairwise overlapping genes were removed from the comparator pathway before correlation analysis, which reduces but does not eliminate correlations arising from shared transcriptional structure, co-regulation, or related biological processes. Random-effects meta-analysis improves cross-cohort synthesis but cannot establish that one pathway regulates another.

#### 6.3.4 Absence of biochemical and structural validation

Finally, mRNA abundance does not capture the defining biochemical and structural events of disulfidptosis. The present analysis provides no direct measurement of the NADPH/NADP^+^ ratio, cystine-to-cysteine reduction, GSH/GSSG balance, protein-disulfide accumulation, actin-disulfide cross-linking, or F-actin collapse. The transcriptomic results should therefore be viewed as hypothesis-generating evidence that prioritises candidate processes for subsequent investigation, rather than as confirmation that disulfidptosis occurs in cardiomyocytes or failing myocardium.

## 7 Current evidence and future experimental validation

Although disulfidptosis has attracted increasing attention in cardiovascular research, direct evidence demonstrating its occurrence in HF remains limited. The currently available evidence can be classified into three levels. First, bioinformatic studies have reported altered expression of disulfidptosis-related genes in cardiovascular diseases, including acute myocardial infarction, ischemic cardiomyopathy, dilated cardiomyopathy, and hypertrophic cardiomyopathy (13, 15, 17, 81). These findings suggest a potential association between disulfidptosis-related molecular signatures and cardiac pathological remodeling. Second, indirect mechanistic evidence indicates that HF is characterized by metabolic remodeling, mitochondrial dysfunction, impaired NADPH-generating capacity, redox imbalance, and cytoskeletal disorganization, all of which may favor a cellular state compatible with a disulfidptosis-like injury (22, 29). Third, and most importantly, direct experimental evidence is required to establish disulfidptosis as a genuine mechanism of cardiomyocyte death. Such evidence should demonstrate SLC7A11-dependent cystine burden, NADPH depletion, thiol-disulfide imbalance, protein disulfide accumulation, actin disulfide cross-linking, F-actin collapse, and rescue by reducing agents or restoration of NADPH-generating capacity (7, 8).

At present, this third level of evidence remains insufficient for HF, making cardiomyocyte-specific validation a priority for future research. *In vitro* studies can use neonatal rat ventricular cardiomyocytes (82), HL-1 cells, AC16 cells (83), and human induced pluripotent stem cell-derived cardiomyocytes exposed to HF-relevant stressors, including glucose restriction, hypoxia/reoxygenation, Ang II stimulation, mechanical stretch, high glucose, palmitate, or doxorubicin (84–87). These models would help determine whether HF-related stress can induce NADPH depletion, GSH/GSSG imbalance, cystine/cysteine disequilibrium, protein disulfide accumulation, and F-actin disruption (28).

*In vivo* validation should also be pursued using established HF models, including transverse aortic constriction, myocardial infarction, ischemia/reperfusion injury, Ang II-induced cardiac remodeling, diabetic cardiomyopathy, and doxorubicin-induced cardiotoxicity (88–91). These models could be used to examine whether failing myocardium exhibits SLC7A11 dysregulation, NADPH insufficiency, thiol-disulfide imbalance, protein disulfide accumulation, and cytoskeletal disorganization (28, 92). Cardiomyocyte-specific manipulation of SLC7A11 or NADPH-generating enzymes would be particularly informative for determining whether disulfidptosis contributes causally to cardiac dysfunction, fibrosis, and ventricular remodeling (22).

Cell-type-resolved validation should accompany these mechanistic studies. Single-cell or single-nucleus RNA sequencing may be used to determine whether SLC7A11/SLC3A2 expression and the proposed redox–cytoskeletal modules are enriched in cardiomyocytes, fibroblasts, endothelial cells, or immune populations across HF aetiologies and disease stages. Spatial transcriptomics or multiplexed *in situ* approaches could further establish whether these signals localise to fibrotic, ischaemic, or remodelling myocardial regions.

Particular attention should be given to cardiac fibroblasts. Recent human single-cell evidence indicates that ferroptosis-associated transcriptional remodelling is enriched in activated fibroblasts in dilated cardiomyopathy. Because fibroblasts undergo marked metabolic and redox changes during matrix remodelling, they may contribute to the bulk SLC7A11–GSH–NADPH-related signal observed in HF tissue. Nevertheless, transcriptomic localisation alone would remain insufficient to establish disulfidptosis. Cell-type-specific validation should combine metabolic measurements, protein-disulfide detection, F-actin imaging, and rescue experiments in purified or genetically defined cardiomyocyte and fibroblast populations. Finally, pathway specificity should be carefully addressed. Because HF involves multiple forms of regulated cell death, future studies need to distinguish disulfidptosis from ferroptosis, apoptosis, pyroptosis, and necroptosis (75, 77). This distinction could be achieved by combining pathway-specific inhibitors with lipid peroxidation assays, caspase activity measurements, MLKL phosphorylation analysis, inflammasome marker assessment, F-actin staining, and rescue experiments aimed at restoring reducing capacity (76, 93, 94). Only when disulfide stress-induced cytoskeletal collapse is shown to occur independently of other death pathways can disulfidptosis be considered a validated mechanism of cardiomyocyte injury in HF. A staged experimental framework for establishing pathway dependence, cytoskeletal involvement, rescue, and specificity is summarized in Table 3. Candidate strategies for testing or modulating reducing capacity, SLC7A11-dependent cystine uptake, NADPH supply, Trx signalling, and cytoskeletal stability, together with their evidence classification and major limitations, are summarized in Table 4. These approaches should be regarded as mechanistic probes or hypothesis-driven interventions rather than established anti-disulfidptosis therapies for HF.

**Table 3:** Proposed experimental validation framework for disulfidptosis in HF.

Validation level	Key question	Suggested models	Required readouts	Expected evidence
Cellular validation	Can HF-related stress induce disulfidptosis-like injury in cardiomyocytes?	NRVMs, HL-1, AC16, hiPSC-CMs treated with glucose restriction, hypoxia/reoxygenation, Ang II, palmitate, high glucose, mechanical stretch, doxorubicin	Cell viability, NADPH/NADP^+^, GSH/GSSG, cystine/cysteine ratio, ROS, protein disulfide burden	HF-related stress induces disulfide stress and cardiomyocyte injury
SLC7A11 dependence	Is the injury dependent on cystine uptake?	SLC7A11 knockdown/overexpression or pharmacological modulation	SLC7A11 expression, cystine uptake, NADPH consumption, protein disulfide accumulation	SLC7A11 modulation changes susceptibility to disulfide stress
Cytoskeletal evidence	Does disulfide stress cause F-actin collapse?	Cardiomyocytes under NADPH-limited stress	Phalloidin staining, confocal imaging, actin fractionation, non-reducing western blot, redox proteomics	Actin disulfide cross-linking and F-actin disorganization are detected
Rescue experiments	Can reducing capacity rescue the phenotype?	DTT, NAC, GSH restoration, NADPH-restoring interventions, PPP activation	Reduced disulfide burden, restored F-actin organization, improved cell survival	Rescue supports causality
Animal validation	Does this occur in failing myocardium?	TAC, MI, I/R, Ang II, diabetic cardiomyopathy, doxorubicin cardiotoxicity	Lipid peroxidation, caspase activation, MLKL phosphorylation, F-actin collapseburden, F-actin integrity	Disulfidptosis-like signatures appear in failing myocardium
Specificity analysis	Is it distinct from other death pathways?	Combined use of ferroptosis, apoptosis, necroptosis inhibitors	Lipid peroxidation, caspase activation, MLKL phosphorylation, F-actin collapse	Disulfidptosis-specific phenotype is distinguished

**Table 4:** Candidate strategies relevant to the proposed disulfidptosis framework in heart failure and their evidence status.

Strategy or agent	Proposed effect within the framework	Evidence in cardiac or HF systems	Evidence classification	Relevance to disulfidptosis	Major limitation	Ref.
NRF2–PPP activation	Promotes antioxidant gene expression and glucose flux through the PPP, thereby supporting NADPH generation and thiol-reducing systems.	NRF2 activation has induced cardiac metabolic reprogramming, enhanced PPP activity, and reduced dysfunction or stress in experimental cardiac models.	Cardiac preclinical evidence.	May increase the reducing capacity required to prevent cystine-associated disulfide stress.	Cardiac protection was not demonstrated to result from inhibition of disulfidptosis; NRF2 also regulates ferroptosis and multiple antioxidant pathways.	(22, 47)
N-acetylcysteine and exogenous thiol support	Provides cysteine or reducing equivalents, supports GSH synthesis, and may decrease protein disulfide accumulation.	NAC has shown protective effects in stressed cardiomyocytes and other cardiac injury settings.	Cardiac mechanistic evidence; non-specific antioxidant intervention.	Suitable for testing whether restoration of thiol-reducing capacity reverses protein-disulfide and F-actin abnormalities.	NAC affects ROS, GSH, PI3K–AKT signalling, mitochondrial function, and several cell-death pathways; rescue would not by itself prove disulfidptosis.	(95)
GSH supplementation or GSH-restoring approaches	Reinforces glutathione-dependent redox buffering and may reduce abnormal protein-disulfide formation.	GSH deficiency and disturbed GSH/GSSG balance have been reported in human and experimental HF, but direct therapeutic evidence in HF disulfidptosis is lacking.	HF-associated redox evidence; intervention largely mechanistic.	May be used as a rescue approach in cellular validation studies.	GSH also suppresses ferroptosis through GPX4 and has broad antioxidant effects; pathway specificity is limited.	(25, 26, 58)
SGLT2 inhibitors	May improve cardiac substrate utilisation, mitochondrial ATP production, and myocardial redox balance, indirectly preserving reducing capacity.	Empagliflozin improved myocardial energy status in diabetic models, and canagliflozin altered human myocardial redox signalling.	Preclinical and human cardiac evidence.	May reduce metabolic and redox conditions considered permissive for disulfidptosis-like injury.	No evidence shows that the clinical benefits of SGLT2 inhibitors are mediated by direct inhibition of disulfidptosis.	(96, 97)
Hydrogen sulfide donors or H_2_S-enhancing approaches	Supports NRF2–SLC7A11–GSH signalling, limits oxidative injury, and may preserve cellular reducing capacity.	H_2_S protected cardiomyocytes from doxorubicin-associated ferroptosis and attenuated ischaemia-induced HF in experimental models.	Cardiac preclinical evidence, primarily ferroptosis- and redox-related.	May be evaluated as a reducing-capacity rescue strategy.	Existing cardiac effects are not specific to disulfidptosis and may involve vasodilation, mitochondrial protection, NRF2 activation, and ferroptosis inhibition.	(55, 98)
SLC7A11 upregulation or cardiomyocyte-specific overexpression	Increases cystine uptake, cysteine availability, GSH synthesis, and resistance to ferroptosis when reducing capacity is sufficient.	Cardiac studies indicate that SLC7A11 can protect against ferroptosis, cardiac hypertrophy, and doxorubicin-related injury.	Cardiac preclinical evidence for ferroptosis regulation.	Useful for determining whether SLC7A11 is protective or detrimental under different NADPH states.	Under NADPH-limited conditions, increased cystine uptake could theoretically increase the cystine-reduction burden; it is not a general anti-disulfidptosis treatment.	(54, 55, 59)
System Xc^−^ inhibition with erastin or sulfasalazine	Reduces cystine uptake and may decrease the NADPH-consuming cystine-reduction burden.	Relevant mechanistic evidence is derived predominantly from tumour or other non-cardiac systems.	Non-cardiac extrapolation; experimental probe.	Can test whether injury depends on SLC7A11-mediated cystine uptake.	These agents may induce ferroptosis by reducing cysteine and GSH availability; they are not established HF therapies and could aggravate myocardial injury.	(7, 8, 56, 57)
Genetic SLC7A11 knockdown or conditional deletion	Determines whether cystine uptake is required for NADPH depletion, disulfide accumulation, and cytoskeletal injury.	Cardiac SLC7A11 studies primarily concern ferroptosis; direct genetic testing in myocardial disulfidptosis has not been reported.	Experimental validation strategy.	Provides stronger causal evidence than pharmacological system Xc^−^ inhibition.	Reduced SLC7A11 may increase ferroptotic susceptibility, requiring parallel lipid-peroxidation and GPX4 measurements.	(54, 59)
PPP or G6PD enhancement	Increases NADPH generation and supports GSH- and Trx-dependent reduction of cystine and protein disulfides.	Cardiac studies support the importance of PPP activity and NADPH generation in adaptation to oxidative and haemodynamic stress.	Cardiac metabolic evidence; proposed disulfidptosis rescue.	Restoration of NADPH generation is a central rescue criterion for testing the proposed mechanism.	Effects of G6PD and PPP activation are model- and disease-stage-dependent; no direct HF disulfidptosis intervention study is available.	(22, 47, 48)
Thioredoxin/TXNRD1 system enhancement	Uses NADPH to maintain protein thiols and reduce abnormal disulfide bonds.	Altered myocardial Trx-system activity has been documented in human ischaemic cardiomyopathy and failing myocardium.	Human cardiac pathway evidence; intervention unvalidated.	Could test whether reinforcement of protein-disulfide repair prevents actin cross-linking and cytoskeletal collapse.	No established intervention has demonstrated that increasing Trx/TXNRD1 activity prevents disulfidptosis in HF.	(25, 33)
Selenium supplementation	May support selenoprotein-dependent antioxidant enzymes, including thioredoxin reductases and GPX4.	General redox and ferroptosis-related rationale exists, but direct HF disulfidptosis evidence is lacking.	Mechanistic extrapolation.	May influence both protein-disulfide repair and ferroptosis defence.	Effects on GPX4 and ferroptosis make pathway attribution difficult; it is not a specific anti-disulfidptosis strategy.	(20, 25)
ROCK inhibition with fasudil or Y-27632	Reduces actomyosin tension and may limit maladaptive cytoskeletal remodelling.	RhoA–ROCK and related mechanosensitive pathways are implicated in cardiac hypertrophy and cytoskeletal remodelling, but these inhibitors have not been shown to prevent myocardial disulfidptosis.	Cardiac pathway rationale; experimental pharmacological probe.	May help determine whether actomyosin tension modifies susceptibility to F-actin collapse.	ROCK inhibition also alters hypertrophy, fibrosis, vascular tone, adhesion, and migration independently of disulfide stress.	(39–43, 51–53)
Actin-polymerisation or myosin inhibitors, including CK-666 and blebbistatin	Experimentally modifies WAVE–Arp2/3-dependent actin polymerisation or actomyosin contraction.	Evidence relevant to disulfidptosis derives mainly from non-cardiac mechanistic models.	Non-cardiac experimental probe.	May clarify whether actin dynamics or contractile tension are required for cytoskeletal collapse.	These compounds are research tools rather than candidate HF treatments and may directly impair cardiomyocyte contraction.	(7, 36)
Direct reducing agents such as DTT	Reduces abnormal protein disulfide bonds and may reverse disulfide-associated cytoskeletal contraction.	Rescue by reducing agents is a defining experiment in established non-cardiac disulfidptosis models.	Non-cardiac mechanistic rescue tool.	Useful as a positive-control rescue intervention for pathway validation.	Not suitable as a therapeutic strategy; broad reduction of protein disulfides lacks cellular and pathway specificity.	(7, 8)
Ferrostatin-1, liproxstatin-1, or iron chelation	Suppresses iron-dependent lipid peroxidation rather than protein-disulfide stress.	Ferroptosis-directed interventions have shown protection in multiple cardiac injury models.	Cardiac ferroptosis evidence; pathway-discrimination control.	Failure of ferroptosis-directed rescue, together with successful thiol/NADPH rescue, would support mechanistic distinction from ferroptosis.	These agents do not directly test or inhibit disulfidptosis and should not be classified as anti-disulfidptosis therapies.	(54, 55, 91, 93)
NAD^+^-restoring or mitochondrial-supportive approaches	May improve mitochondrial metabolism and indirectly preserve cellular redox and NADPH homeostasis.	Cardiac metabolic benefits have been reported for several mitochondrial or NAD^+^-related approaches, but their relationship to myocardial disulfide stress is indirect.	General cardiac metabolic evidence; conceptual extrapolation.	Could reduce the metabolic conditions considered permissive for disulfidptosis-like injury.	NAD^+^ abundance is not equivalent to NADPH availability, and improved mitochondrial function does not demonstrate disulfidptosis inhibition.	(45, 48, 61, 62)

“Cardiac preclinical evidence” denotes findings obtained in cultured cardiomyocytes or animal models of cardiac injury or HF. “Human cardiac evidence” denotes observations from human myocardial tissue or clinical cardiac samples. “Non-cardiac extrapolation” denotes mechanisms established primarily in tumour or other non-cardiac systems. “Experimental probe” denotes an intervention used to test pathway dependence and should not be interpreted as a therapeutic candidate. None of the listed interventions has been demonstrated to improve HF through direct inhibition or modulation of disulfidptosis.

DTT, dithiothreitol; G6PD, glucose-6-phosphate dehydrogenase; GPX4, glutathione peroxidase 4; GSH, reduced glutathione; GSSG, oxidised glutathione; H_2_S, hydrogen sulfide; HF, heart failure; NAC, N-acetylcysteine; NADPH, reduced nicotinamide adenine dinucleotide phosphate; NRF2, nuclear factor erythroid 2-related factor 2; PPP, pentose phosphate pathway; ROCK, Rho-associated protein kinase; SGLT2, sodium–glucose cotransporter 2; Trx, thioredoxin; TXNRD1, thioredoxin reductase 1.

## 8 Discussion: controversies and knowledge gaps

Previous cardiovascular studies have approached disulfidptosis predominantly through differential-expression analysis, diagnostic modelling, immune-infiltration assessment, and computational target prediction. These studies are informative for identifying disease-associated transcriptional patterns, but they do not establish whether the canonical disulfidptosis cascade is active in the failing heart. The contribution of the present review lies not in identifying additional signature genes, but in reframing disulfidptosis as a testable problem of metabolic–redox–cytoskeletal coupling in HF. The proposed framework connects impaired NADPH-generating capacity and the SLC7A11-dependent cystine-reduction burden with thiol–disulfide disequilibrium, actin-network disruption, and myocardial remodelling. It therefore distinguishes mechanistic compatibility from demonstrated cell death.

Within this framework, the transcriptomic findings should be regarded as contextual evidence rather than confirmation of pathway activity. Their purpose is to determine whether genes representing the proposed metabolic, redox, and cytoskeletal modules are co-altered in bulk failing myocardium, not to infer NADPH depletion, protein disulfide accumulation, or F-actin collapse from mRNA abundance. Establishing disulfidptosis in HF will require convergent biochemical and structural evidence, including an SLC7A11-dependent cystine burden, depletion of reducing equivalents, accumulation of protein disulfides, actin disulfide cross-linking, and cytoskeletal collapse, together with rescue following restoration of NADPH supply or thiol-reducing capacity. Cell-type-resolved analyses will also be necessary to determine whether the observed signals originate in cardiomyocytes or non-myocyte compartments. This distinction is particularly important because recent single-cell analysis of human dilated cardiomyopathy identified a ferroptosis-associated redox programme predominantly in fibroblasts, where it was linked to fibroblast activation and profibrotic remodelling (99). Given the shared SLC7A11–GSH–NADPH node, stromal cells may contribute to the bulk disulfidptosis-related transcriptional patterns observed in HF. However, this overlap should not be interpreted as evidence that fibroblasts undergo disulfidptosis. Rather, it identifies fibroblasts as an additional cell population in which the metabolic, redox, and cytoskeletal requirements of disulfidptosis should be tested directly. Several key questions remain. First, it is unclear whether cardiomyocytes can reach the level of reducing-capacity failure required to trigger disulfidptosis. Adult cardiomyocytes possess multiple NADPH-generating systems that may buffer against disulfide stress, especially under physiological or early compensatory conditions. In advanced HF, however, mitochondrial dysfunction, metabolic inflexibility, oxidative stress, and impaired substrate utilization may weaken this buffering capacity. Second, the role of SLC7A11 in failing myocardium is likely context dependent. When NADPH supply is sufficient, SLC7A11-mediated cystine uptake may support glutathione synthesis and protect against oxidative stress and ferroptosis. Under NADPH-limited conditions, however, sustained cystine uptake could increase the cystine reduction burden and promote disulfide accumulation. Thus, SLC7A11 should not be simply defined as either protective or detrimental in HF.

A central challenge is to distinguish disulfidptosis from ferroptosis, because both pathways converge on the SLC7A11–GSH–NADPH regulatory node. Ferroptosis nevertheless has a more developed HF-specific evidence base. Iron-dependent lipid peroxidation, failure of the GSH–GPX4 defence system, and pharmacological rescue have been reported across pressure-overload, ischaemic, diabetic, septic, obesity-related, and doxorubicin-induced cardiac injury models (60). By comparison, direct evidence for disulfidptosis in cardiomyocytes or failing myocardium remains unavailable.

This difference in evidence maturity should be reflected in pathway interpretation. Changes in SLC7A11 expression, GSH availability, NADPH status, or general oxidative stress cannot by themselves distinguish the two processes. Ferroptosis requires evidence of iron-dependent phospholipid peroxidation and its suppression by ferroptosis-directed interventions, whereas disulfidptosis requires demonstration of protein disulfide accumulation, actin disulfide cross-linking, F-actin collapse, and rescue through restoration of reducing capacity. Until these criteria are met, SLC7A11–GSH–NADPH alterations in HF should be interpreted as changes in a shared redox-regulatory node rather than as evidence of a specific cell-death programme. It also remains unclear whether disulfidptosis is broadly relevant to HF or restricted to specific subtypes. This mechanism may be more likely to emerge in conditions marked by severe metabolic stress, redox failure, and cytoskeletal instability, such as ischemic HF, diabetic cardiomyopathy, pressure overload-induced HF, doxorubicin-induced cardiotoxicity, and metabolically driven HF with preserved ejection fraction. Comparative studies across HF etiologies and disease stages will be needed to clarify this issue.

From a translational perspective, interventions targeting disulfidptosis remain premature. Potential strategies such as N-acetylcysteine, sodium-glucose cotransporter 2 inhibitors, hydrogen sulfide donors, NADPH-restoring approaches, SLC7A11 modulation, and cytoskeleton-protective interventions may offer future directions, but none has been shown to improve HF by directly inhibiting disulfidptosis (95–98). These approaches should therefore be regarded as exploratory therapeutic avenues rather than established anti-disulfidptosis treatments.

In conclusion, disulfidptosis offers a theoretical perspective for understanding HF-related myocardial injury by linking metabolic insufficiency, impaired reducing capacity, thiol-disulfide imbalance, and cytoskeletal collapse. Direct evidence in cardiomyocytes and failing myocardium remains limited, and future studies should determine whether disulfidptosis represents an independent form of cardiomyocyte death, a component of broader redox-related injury, or a context-dependent mechanism restricted to specific HF phenotypes.
