# Supplementary material for: Disulfidptosis in heart failure: an emerging mechanism awaiting exploration
Source: Front Cardiovasc Med. 2026 Sep 9;13:1842464. doi: 10.3389/fcvm.2026.1842464 (PMC13598392; doi:10.3389/fcvm.2026.1842464)
Supplement: Supplementary file 1 [file Table1.docx]

Supplementary Information

| **Feature** | **Disulfidptosis (Predicted)** | **Ferroptosis** | **Apoptosis** | **Necroptosis** | **Autophagy** | **Pyroptosis** |
| --- | --- | --- | --- | --- | --- | --- |
| Dependence on NADPH | key trigger | – | – | – | – | – |
| Dependence on lipid peroxidation | – | core mechanism | – | – | – | – |
| Caspase involvement | – | – | Caspase-3/8/9 | – | – | Yes (Caspase-1/4/5/11) |
| F-actin collapse | hallmark feature | – | – | – | – | – |
| Type of ROS | Disulfide stress | Lipid peroxidation ROS | Mitochondrial ROS | Inflammatory ROS | ROS can induce but not required | Inflammatory ROS |
| Key regulatory molecules | SLC7A11, INF2, Actin Cys374 | GPX4, ACSL4, SLC7A11 | Bax, Bcl-2, Caspases | RIPK1, RIPK3, MLKL | Beclin1, LC3, ATG5/7, mTOR | NLRP3, ASC, GSDMD, Caspase-1 |
| Typical inhibitors | DTT, β-mercaptoethanol | Ferrostatin-1, Liproxstatin-1 | Z-VAD-FMK | Necrostatin-1 | 3-MA, Bafilomycin A1 | MCC950, VX-765 |
| Mitochondrial changes | Not typical | Mitochondrial shrinkage | Cytochrome-c release | Mitochondrial damage | Mitochondria degraded by autophagy (mitophagy) | Mitochondrial damage |
| Cellular morphological features | F-actin collapse | Mitochondrial cristae shrinkage | Nuclear condensation, DNA fragmentation | Cell swelling | Autophagosome formation | Cell swelling, membrane pore formation |

**Supplementary Table S1.** Comparison of molecular features between disulfidptosis and other regulated cardiomyocyte death pathways in heart failure
